# Ultrasmall Copper Nanoclusters in Zirconium Metal‐Organic Frameworks for the Photoreduction of CO_2_

**DOI:** 10.1002/anie.202211848

**Published:** 2022-09-23

**Authors:** Shan Dai, Takashi Kajiwara, Miyuki Ikeda, Ignacio Romero‐Muñiz, Gilles Patriarche, Ana E. Platero‐Prats, Alexandre Vimont, Marco Daturi, Antoine Tissot, Qiang Xu, Christian Serre

**Affiliations:** ^1^ Institut des Matériaux Poreux de Paris Ecole Normale Supérieure ESPCI Paris CNRS PSL University 75005 Paris France; ^2^ AIST-Kyoto University Chemical Energy Materials Open Innovation Laboratory (ChEM-OIL) National Institute of Advanced Industrial Science and Technology (AIST) Sakyo-ku, Kyoto 606-8501 Japan; ^3^ Normandie Univ. ENSICAEN UNICAEN CNRS Laboratoire Catalyse et Spectrochimie 14000 Caen France; ^4^ Departamento de Química Inorgánica Facultad de Ciencias Universidad Autónomade Madrid Campus de Cantoblanco 28049 Madrid Spain; ^5^ Condensed Matter Physics Center (IFIMAC) Universidad Autónoma de Madrid Campus de Cantoblanco 28049 Madrid Spain; ^6^ Instituto de Investigación Avanzada en Ciencias Químicas de la UAM Universidad Autónoma de Madrid Campus de Cantoblanco 28049 Madrid Spain; ^7^ Université Paris-Saclay CNRS Centre de Nanosciences et de Nanotechnologies 91120 Palaiseau France; ^8^ Institute for Integrated Cell-Material Sciences (iCeMS) Kyoto University Sakyo-ku, Kyoto 606-8501 Japan; ^9^ Shenzhen Key Laboratory of Micro/Nano-Porous Functional Materials (SKLPM) Department of Chemistry and Department of Materials Science and Engineering Southern University of Science and Technology (SUSTech) Nanshan, Shenzhen Guangdong 518055 China

**Keywords:** CO_2_ Reduction, Core-Shell Composites, Photocatalysis, Zr-MOFs, In Situ Spectroscopies

## Abstract

Encapsulating ultrasmall Cu nanoparticles inside Zr‐MOFs to form core–shell architecture is very challenging but of interest for CO_2_ reduction. We report for the first time the incorporation of ultrasmall Cu NCs into a series of benchmark Zr‐MOFs, without Cu NCs aggregation, via a scalable room temperature fabrication approach. The Cu NCs@MOFs core–shell composites show much enhanced reactivity in comparison to the Cu NCs confined in the pore of MOFs, regardless of their very similar intrinsic properties at the atomic level. Moreover, introducing polar groups on the MOF structure can further improve both the catalytic reactivity and selectivity. Mechanistic investigation reveals that the Cu^I^ sites located at the interface between Cu NCs and support serve as the active sites and efficiently catalyze CO_2_ photoreduction. This synergetic effect may pave the way for the design of low‐cost and efficient catalysts for CO_2_ photoreduction into high‐value chemical feedstock.

## Graphical abstract

A room‐temperature synthetic strategy is reported for incorporation of ultrasmall Cu nanoclusters into a series of robust Zr‐metal–organic frameworks. The resultant core–shell composites mediate CO_2_ photoreduction selectively and outperform similar composites even with variable Cu spatial distribution.

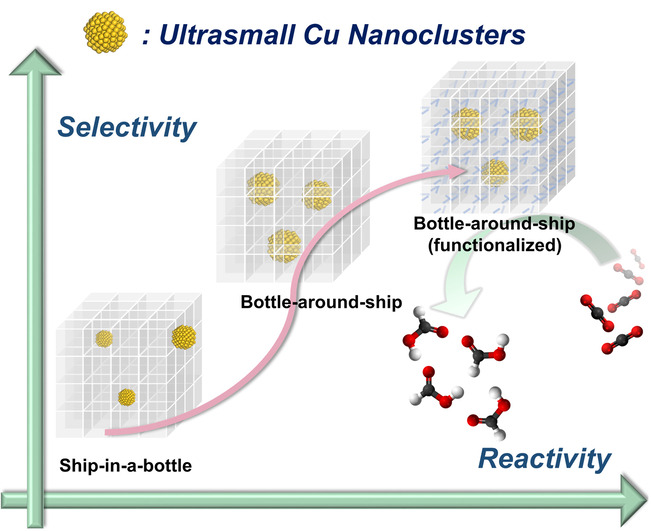

## Introduction

The massive emission of CO_2_ into the atmosphere is considered a hazard nowadays. One of the most promising techniques to overcome this threat is to use catalytically reactive porous materials to trap and convert CO_2_ into chemical feedstocks.^[1]^ Previous works have focused on CO_2_ reduction by hydrogenation at high temperatures regardless of the additional cost of hydrogen.^[2]^ Electrocatalytic CO_2_ reduction is another promising alternative, even though it might give rise to the decomposition of the resulting chemicals due to the commonly required overpotential and additional electricity needs, which is a secondary form of energy.^[3]^ Consequently, the photoreduction of CO_2_ has been drawing considerable attention due to the abundance and renewability of solar energy.

Cu nanoclusters (NCs) are a class of ultrasmall metallic nanoparticles (NPs), typically with a size lower than 2 nm.^[4]^ The well‐known size‐dependent catalytic effect makes Cu NCs more appealing compared to larger sized Cu nanoparticles due to the increased surface area of active atoms.^[5]^ Moreover, Cu is an inexpensive metal with a large abundance, in contrast with noble‐metal‐based photocatalysts that have been widely studied, and can selectively reduce CO_2_ to CO.^[6]^ In addition, Cu can lead to CO_2_ reduction into hydrocarbon products, including C1 to C2, which is advantageous from the industrial point of view.[^5a^, ^7^] However, Cu NCs are very fragile due to their ultrasmall size and therefore very high surface energy, and the aggregation of Cu NCs is often unavoidable, particularly in harsh conditions (e.g., high temperature).

Metal–organic frameworks (MOFs) are crystalline hybrid solids that usually show high porosity associated with highly tunable physical/chemical properties for use in targeted catalytic applications.^[8]^ Zr‐based MOFs are a particularly appealing subset of MOFs, presenting in most cases a very high chemical and thermal stability together with an almost infinite structural diversity. Encapsulating catalytic inorganic NPs into Zr‐MOFs has therefore been intensively studied, particularly for the conventional loading of NPs in the cavity of MOFs.^[9]^ However, many issues may occur when using these solids as catalysts, such as the leaching of active NPs, the hindered diffusion of substrate and the undefined spatial distribution of active sites. Constructing a MOF shell around the preformed nanoparticles to form a core–shell structure is one effective method to prevent the above‐mentioned drawbacks.^[10]^ However, a great challenge remains in making core–shell NPs@MOF because of the lattice mismatch between the NPs and the MOF. Exciting achievements have been reported dealing with noble metal NPs, such as Au, Pt, and Pd.[^9a^, ^11^] Unfortunately, attempts to prepare Cu NPs@MOFs remain scarce, probably due to the high activity of Cu NPs that can hamper the synthesis of the targeted structures. Notably, encapsulating ultrasmall Cu NCs into Zr‐MOFs is particularly interesting for CO_2_ reduction as the versatility of Zr‐MOFs may allow many investigation of the catalytic interface effect. Several tentative efforts have been reported recently to develop core–shell structures based on large Cu NPs (≥18 nm) with a reasonable catalytic performance.[^2^, ^12^] However, the formation of ultrasmall Cu nanoclusters (Ø≤2 nm)@MOFs remains an unexplored field. Furthermore, preparing any core–shell NPs@MOFs at gram‐scale is challenging regardless of their usefulness in practical applications.

Herein, we apply a gentle room‐temperature, aqueous‐solution‐based strategy to incorporate, for the first time, ultrasmall Cu nanoclusters in two benchmark microporous Zr‐MOFs (Zr‐fumarate/MOF‐801 and UiO‐66‐NH_2_). The low cost of the raw materials and green synthesis conditions enable us to optimize the synthetic parameters to achieve gram‐scale catalyst preparation. The prepared composites display high reactivity and selectivity for the photoconversion of CO_2_ to chemical feedstock at room temperature. In addition, we show how our method leads to highly reactive solids through a comparison with the lower catalytic reactivity obtained with Cu NCs on the MOFs, the NCs being mainly on the surface or in the pore space of the MOF. Finally, spectroscopy sheds light on the nature of the Cu species with X‐Ray absorption spectroscopy revealing the similar intrinsic properties of Cu nanoclusters with different spatial distributions, in relation with the position‐incurred catalytic activity difference; while *in situ* infrared spectroscopy shows the importance of the Cu^I^ sites stabilized at the surface of NCs.

## Results and Discussion

**Figure 1:**
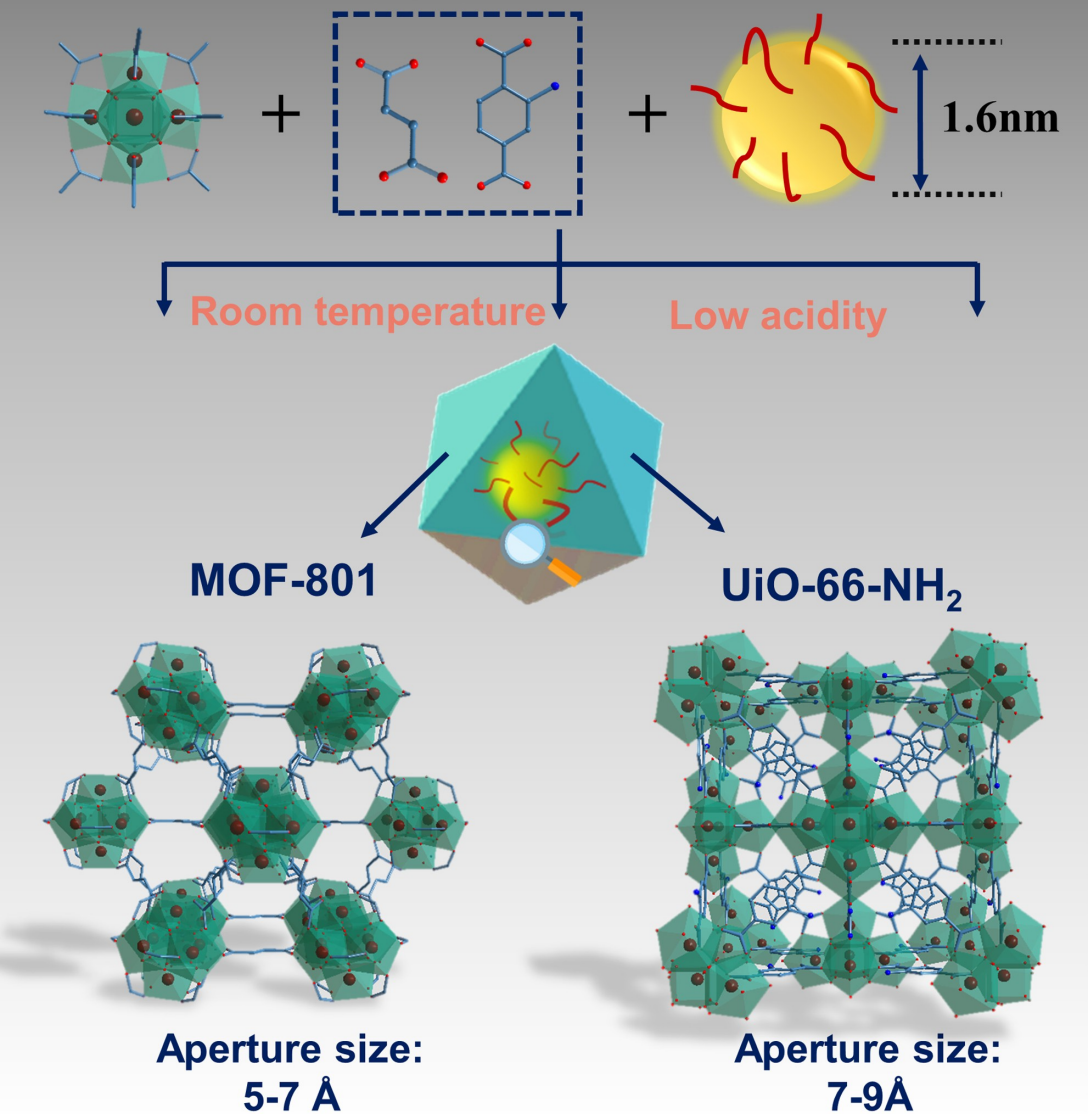
Illustration of the “bottle‐around‐ship” strategy applied in this work, as well as the involved Cu NCs and Zr‐MOFs and their basic information.

**Figure 2:**
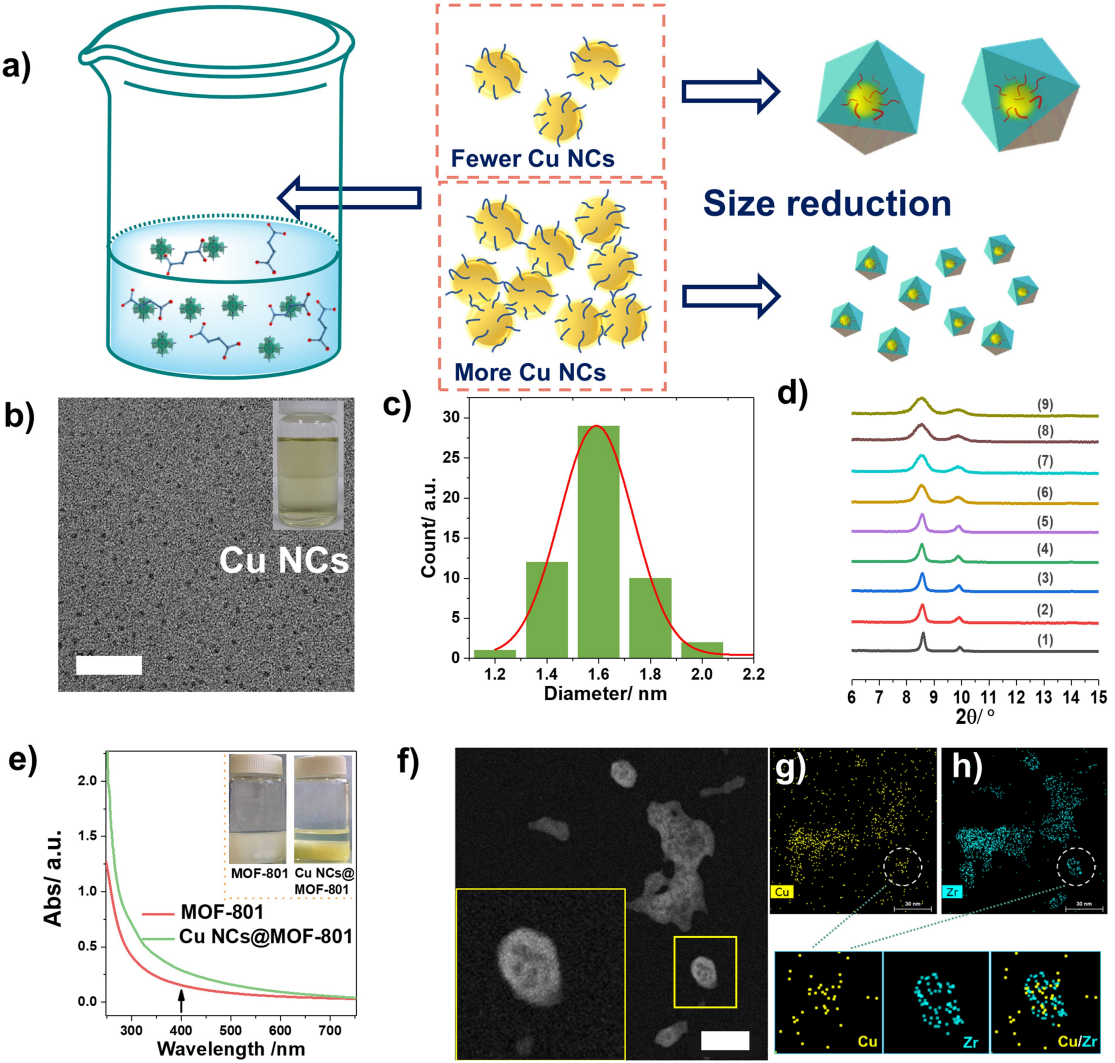
a) Representation of the Cu NCs concentration‐dependent size reduction of the prepared Cu NCs@MOF‐801; b) TEM images of Cu NCs; insert: a photograph of the synthesized Cu NCs; c) particle size distribution of the synthesized Cu NCs; d) PXRD patterns (λ_Cu_=1.5406 Å) of MOF‐801(1) and CuNPs@MOF‐801 with increasing Cu NCs loading from 64 μmol to 6.4 mmol (labeled with 1–9); e) solid UV‐vis reflectance spectra of MOF‐801 and Cu NCs@MOF‐801 synthesized with 6.4 mmol Cu NCs; f) The aberration‐corrected high‐angle annular dark‐field scanning transmission electron microscopy (ac‐HAADF‐STEM) image of Cu NCs@MOF‐801 synthesized with 6.4 mmol Cu NCs insert: zoomed‐in image; g,h) EDS elemental (Zr, Cu) mapping of Cu NCs@MOF‐801 with 6.4 mmol Cu NCs. Three smaller images: magnified EDS images; Scale bar in b) and f)=20 nm.

The “bottle‐around‐ship” strategy relies on the fabrication of the MOF shell around pre‐synthesized nanoparticles to form guest@host composites (illustrated in Figure 1). The synthesis of ultrasmall Cu nanoclusters (Cu NCs) was performed using l‐ascorbic acid as both reducing and capping agent thanks to its strong affinity with Cu. High‐resolution transmission electron microscopy (HRTEM; Figure 2b) indicated that the yellowish solution obtained after stirring for 1 h at room temperature contained 1.6 nm monodispersed Cu nanoclusters (Figure 2c). It is documented that ultrasmall Cu NCs are often much less thermally/chemically stable than other common nanoclusters (e.g., Ag, Au, and Pt NCs) due to their higher reactivity that can lead to aggregation and oxidation at elevated temperatures in solution. Unlike most of the robust MOFs (e.g., Zr‐MOFs) that are synthesized using solvo/hydrothermal methods, we applied a novel weakly acidic room‐temperature strategy to design Cu NCs@MOF composites without altering the Cu NCs properties. The synthesis required the use of pre‐synthesized Zr_6_ oxoclusters, which represents a much less acidic starting material in comparison to other Zr salts used in our previous study.^[13]^ Indeed, using ZrCl_4_ as the starting metal salt for the synthesis of Zr‐MOFs led to the total dissolution of Cu NCs, in agreement with reported results.^[2]^ The introduction of Cu NCs in the MOF precursor solution was compatible with the MOF growth even though the preparation was in gram‐scale as proven by both PXRD and 77 K N_2_ isotherm measurements (see Figures S1 and S2). The preservation of porosity suggested that the spatial distribution of Cu NCs is more likely to be core–shell, as opposed to the conventional loading of nanoparticles in the pore space of a given MOF that usually leads to a strong decrease of the porosity. The prepared composites exhibited a larger N_2_ adsorption capacity at 1 bar due to the interparticle mesoporosity, as typically observed for nanomaterials.[^13^, ^14^] Since the size of Cu NCs (1.6 nm) is larger than the aperture of MOF‐801 (5–7 Å), the Cu NCs might also be grafted on the surface of MOF particles due to the affinity between MOF and Cu NCs. In order to assess this, we simply mixed pre‐formed MOF‐801 nanocrystals and Cu NCs solution in the same conditions as the abovementioned “bottle‐around‐ship” strategy. Inductively coupled plasma (ICP) analysis on the collected sample revealed an absence of Cu in the sample, indicating that the Cu NCs are unlikely to be attached on the outer surface of MOF particles. Interestingly, after carefully investigating the relationship between the introduced amount of Cu NCs and the nucleation of MOFs, a dramatic Cu NCs concentration‐dependent MOF Bragg peak broadening was observed (Figure 2d), with a decrease of particle size of Cu NCs@MOF as a function of the introduced NCs quantity (see Figure 2a). SEM and TEM images in Figures S4–S6 (corresponding to PXRD patterns in Figure 2d (1), (2), and (9)) confirmed our hypothesis. These results are in agreement with a nucleation process driven by a seed‐mediated crystal growth, where the small Cu NCs seeds act as nucleation sites and are prevented from growing further as larger crystals. This is in good agreement with previous reports, where the introduced guest nanoparticles led to a downsizing of the MOF shell.^[15]^ A control experiment using one equivalent of l‐ascorbic acid in the synthesis without Cu nanocrystals did not result in a similar downsizing (no PXRD peak broadening, Figure S7), further confirming the seed‐mediated crystal growth. UV‐visible diffuse reflectance spectroscopy was applied to assess the lack of agglomeration of ultrasmall Cu NCs inside the MOF crystals according to the characteristic localized surface plasmon resonance (LSPR) spectrum of larger sized Cu NPs. Here, Cu NCs@MOF‐801 presented a yellowish color (Figure 2e) without a specific plasmonic absorption peak due to the quantum size effect usually observed with ultrasmall inorganic nanoparticles (<3 nm).^[16]^ Moreover, the Cu NCs@MOF‐801 exhibited a greenish color, distinct from the original yellow color after 600 °C annealing under O_2_ (Figure S8), indicating that the initial Cu was likely to be in a reduced form rather than Cu^II^. The aberration‐corrected high‐angle annular dark‐field scanning TEM (ac‐HAADF‐STEM) measurements in Figure 2f hinted at a possible spatial distribution of ultrasmall Cu NCs (the black spots) in the MOF‐801 nanocrystals. The location of Cu NCs was subsequently confirmed by elemental mapping (EDS technique). As shown in Figure 2g–h as well as in the zoomed‐in images, the whole MOF crystal exhibit some “empty” centers, due to the incorporated Cu NCs (yellow dots). Notably, it was almost impossible to localize Cu NCs due to their small size and to the low atomic number of Cu compared to Zr from the MOF shell. Finally, the energy dispersive X‐ray spectroscopy (EDS) indicated that the content of the introduced Cu NCs is 2.8 % in atomic ratio relative to Zr (97.2 %), which is identical to our observation from ICP‐MS analysis (2.85 % Cu).

**Figure 3:**
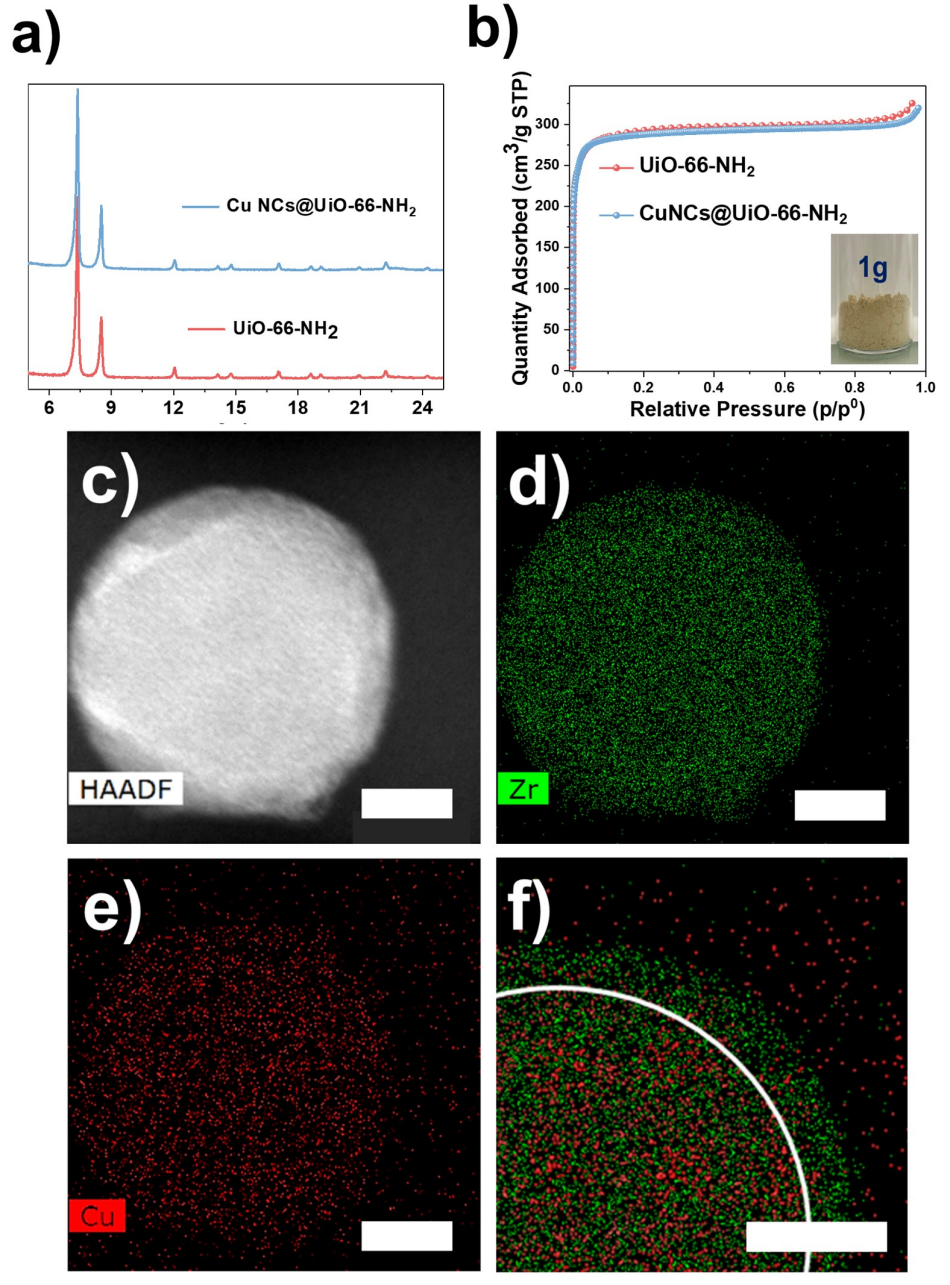
a) PXRD patterns (λ_Cu_=1.5406 Å); b) N_2_ isotherms of synthesized Cu NCs@UiO‐66‐NH_2_ composites and pristine UiO‐66‐NH_2_ (T=77 K, *P*_0_=1 bar); c–f) High‐angle annular dark‐field scanning transmission electron microscopy (HAADF‐STEM) images and corresponding EDS elemental (Zr, Cu, and Zr/Cu) mapping of Cu NCs@UiO‐66‐NH_2_; Scale bar=30 nm.

The large structural diversity of Zr‐MOFs allows for many possibilities to tune the functions of the shell MOFs. For instance, UiO‐66‐NH_2_ is an amino‐functionalized microporous MOF, where the amino groups can effectively improve the light harvesting as well as the affinity with guest molecules like CO_2_. By performing similar room‐temperature syntheses, homologous hybrid composites were obtained. As shown in Figure 3a–b, the synthesized Cu NCs@UiO‐66‐NH_2_ displayed identical crystallinity and porosity compared to pristine UiO‐66‐NH_2_, regardless of the loading of 2.5 at % of Cu. The ambient, facile synthesis allowed us to achieve gram‐scale products without a change in quality (the characterization mentioned here was all based on gram‐scale products). The HAADF‐STEM and EDS mapping in Figure 3c–f demonstrated a similar distribution of the Cu NCs. Notably, seed‐mediated MOF growth was also observed in this case and the size of composite particles decreased from ca. 200 nm to ca. 80 nm (Figures S9 and S10) in the presence of Cu NCs, which is in agreement with our previous findings with Cu NCs@MOF‐801.

**Figure 4:**
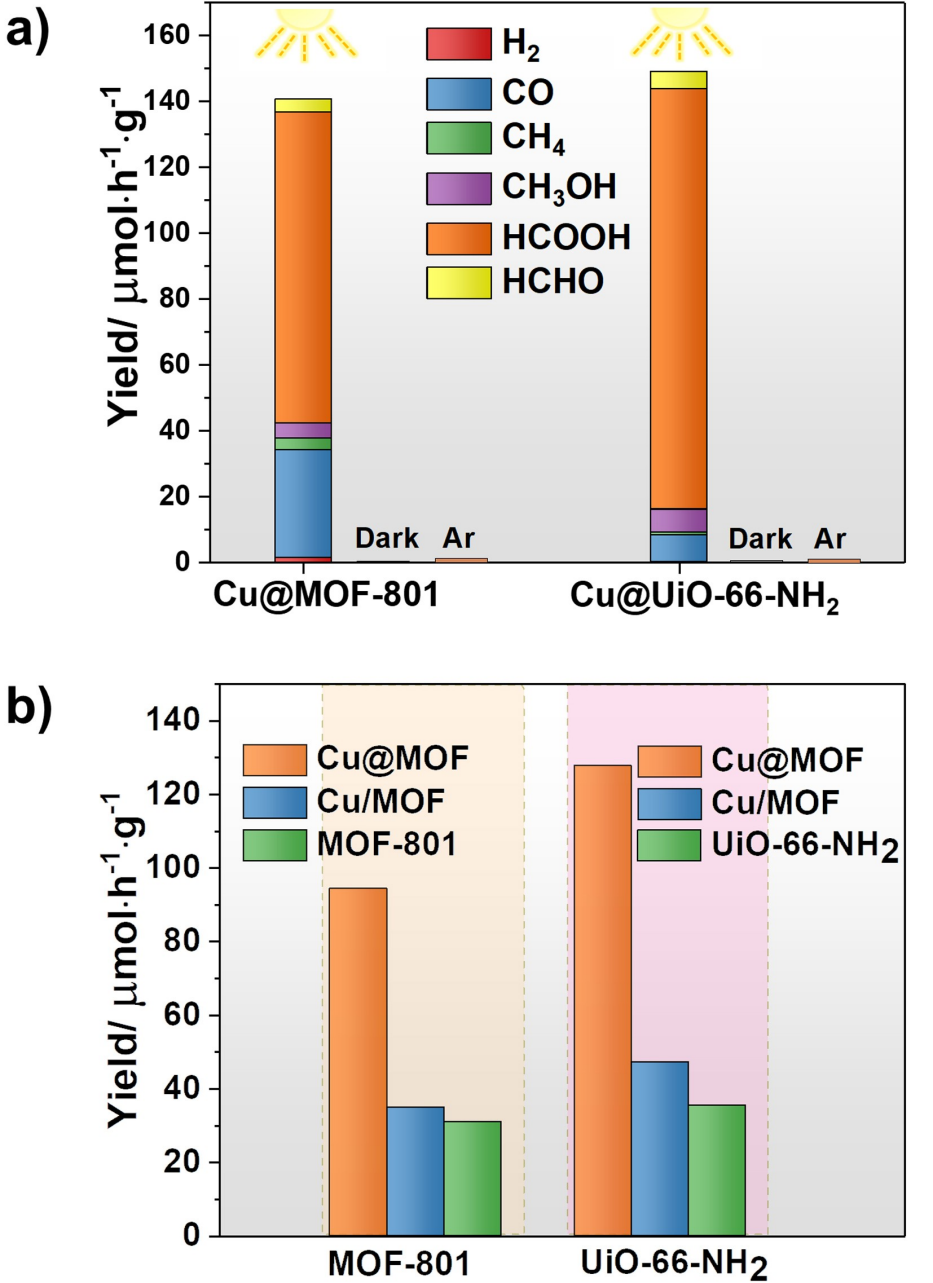
a) Production of the photocatalyzed CO_2_ with Cu NCs@MOF‐801 and Cu NCs@UiO‐66‐NH_2_ compared to the corresponding blank experiments; b) Comparative rates of HCOOH formation with Cu/MOFs, and core–shell Cu NCs@MOFs.

**Figure 5:**
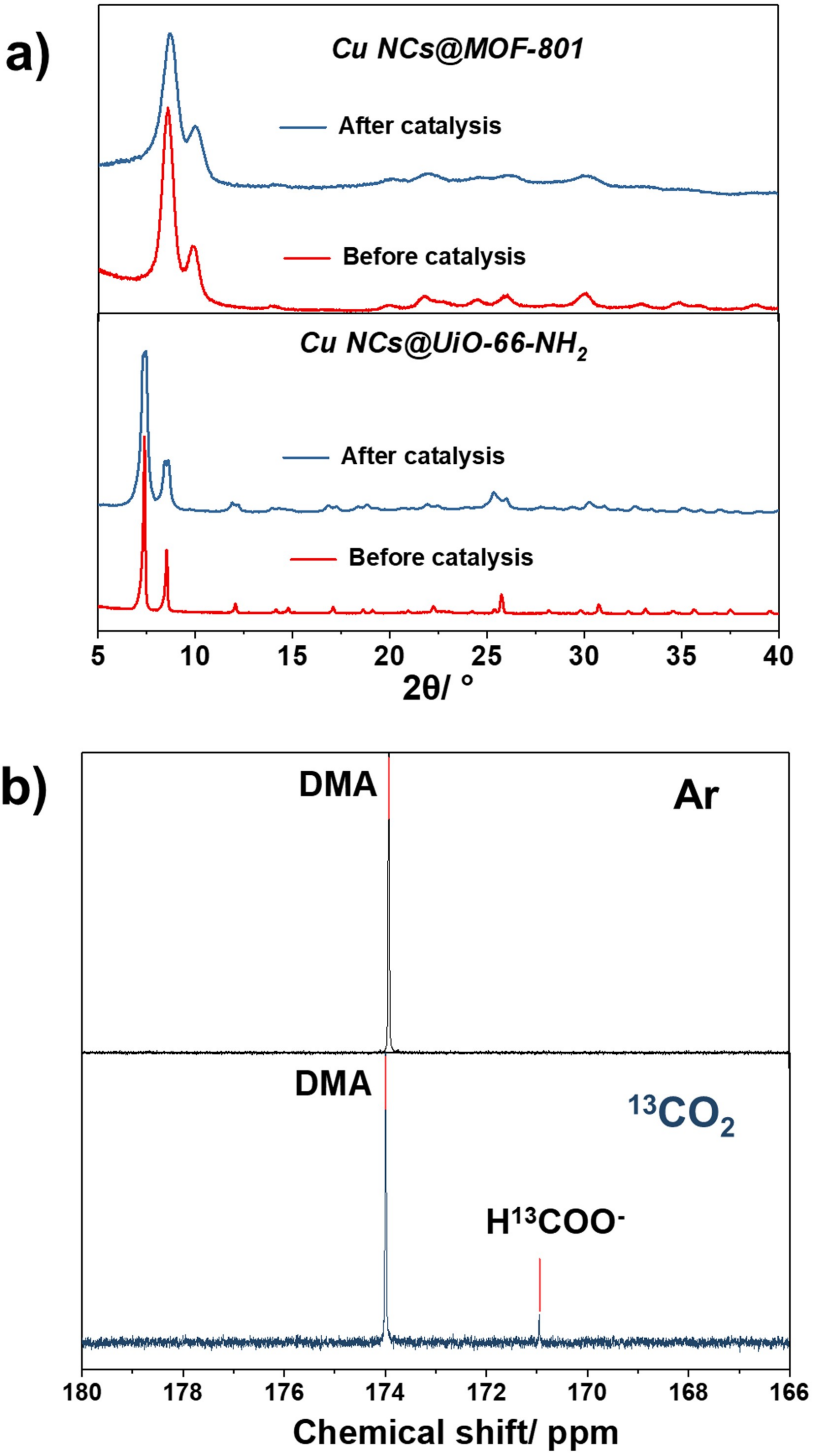
a) PXRD patterns (λ_Cu_=1.5406 Å) of Cu NCs@MOF‐801 and Cu NCs@UiO‐66‐NH_2_ before and after photocatalysis; b) ^13^C NMR spectra of the photocatalysis products under Ar and ^13^CO_2_ gas feeding.

As Cu NCs hold the unique ability to catalyze challenging CO_2_ reduction to organic feedstock, we selected CO_2_ photocatalytic reduction as a model reaction to investigate the core–shell activity and to build structure–property relationships. To the best of our knowledge, photoreduction of CO_2_ to form organic C1 products using core–shell Cu NCs@MOFs composites has unfortunately not been reported so far, mainly due to the difficulty in synthesizing composites. The photoreduction experiments were carried out by using triethanolamine (TEOA) as a sacrificial agent under UV irradiation (385 nm) at room temperature for 18 h. The gas phase and the liquid phase were analyzed by gas chromatography (GC) and liquid chromatography (LC), respectively. In the case of Cu NCs@MOF‐801, CO (22.5 %) and HCOOH (64.9 %) were found as the main products. The evolution rates of CO and HCOOH were about 32 and 94 μmol h^−1^ g^−1^, respectively. The major generation of CO and HCOOH suggested the two‐electron reduction of CO_2_ by the Cu NCs@MOF‐801 catalyst, as either CO or HCOOH only needs two electrons to be produced from CO_2_. In contrast, the core–shell Cu NCs@UiO‐66‐NH_2_ showed an enhanced reactivity for formic acid product, with an evolution rate of 128 μmol h^−1^ g^−1^, demonstrating that the use of UiO‐66‐NH_2_ shell can effectively improve the production of formic acid likely due to the enhanced affinity of this MOF for CO_2_ (NH_2_‐CO_2_ interaction) as proved by CO_2_ sorption isotherm measurements (Figure S11). Interestingly, the selectivity of the formation of HCOOH reached 86 % due to a lower CO production (blue block in Figure 4a). At the same time, the other byproducts (CH_3_OH, CH_4_, and HCHO) were only observed in limited quantities and did not exhibit much change upon varying catalysts, which suggests that the catalytic centers in these two cases are similar. The change in selectivity between CO and HCOOH can be ascribed to the difference in surface functionalization of the shell MOFs. One assumes that the electron‐accepting NH_2_ groups exhibit stronger host–guest interactions with formic acid and/or formates, which might, in turn, facilitate the formation of such species.^[17]^ The high catalytic efficiency here is competitive with many MOF‐based examples regardless of their slightly different conditions (see Table S1) and, furthermore, is advantageous in comparison with most catalysts requiring complex synthesis conditions and/or expensive materials.^[18]^ The stability of the Cu NCs@MOF‐801 and Cu NCs@UiO‐66‐NH_2_ during the catalytic reaction was subsequently evaluated. As displayed in Figure 5a, PXRD patterns of both composites remained unchanged after photocatalysis, indicating the robustness of the structures. Notably, the blank catalytic experiments, including the one under dark conditions and the one with only Ar as the feeding gas, did not lead to any detectable products (Figure 4a), suggesting the strong dependence of the catalysis on both light and CO_2_. In addition, to confirm the origin of the synthesized formates/formic acid moieties, we carried out experiments using ^13^CO_2_ and Ar feeding, respectively. The ^13^C nuclear magnetic resonance (NMR) spectrum in Figure 5b clearly demonstrated that formates/formic acid molecules merely come from CO_2_ rather than the degradation of residual organic species in our case.

In order to understand how the location of the Cu NCs influences the catalytic performance, we mixed Cu ions with the pre‐formed MOF‐801 or UiO‐66‐NH_2_ nanoparticles in the solution and subsequently reduced them with l‐ascorbic acid. We fixed all the synthetic parameters including solvents and precursors while adjusting the quantity of Cu and l‐ascorbic acid to have the same Cu content in the final compound compared to the core–shell composites. The N_2_ adsorption at 77 K revealed a porosity reduction after the introduction of guest Cu NCs (Figure S12). In addition, HRTEM and STEM images in Figures S13 and S14, revealed that the loaded Cu is distributed mostly in the cavity of MOFs or partially at the outer surface of the MOF particle, without any observable large Cu nanoparticles. These composites (labeled Cu/MOF‐801 and Cu/UiO‐66‐NH_2_) both showed dramatically lower reactivity in the formation of HCOOH (2.7 and 2.8 times, respectively, Figure 4b). Therefore, core–shell Cu NCs@MOFs architectures outperformed the traditional Cu NCs loaded in the outer surface or the cavity of MOFs with the two MOF carriers.

**Figure 6:**
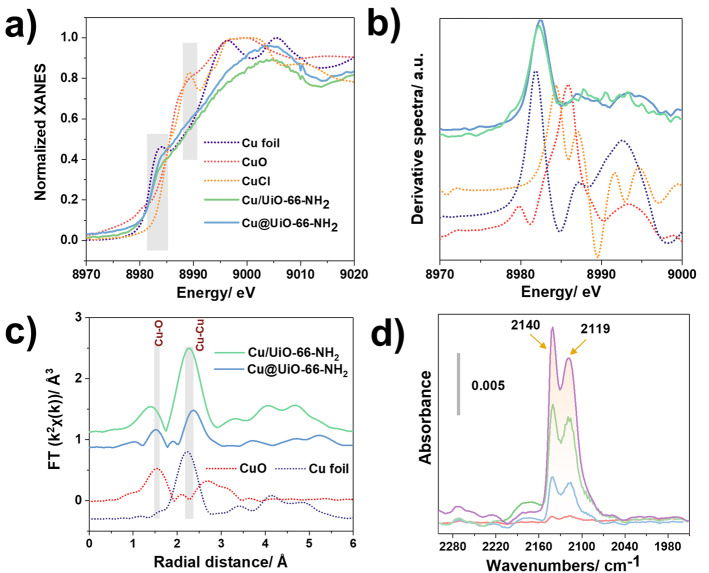
Cu K‐edge a) XANES and b) corresponding derivative spectra of Cu NCs@UiO‐66‐NH_2_, Cu/UiO‐66‐NH_2_, Cu foil, CuCl and CuO; c) Fourier‐transformed EXAFS spectra of Cu composites and references; d) in situ FTIR spectra of CO adsorption at room temperature from small dose to equilibrium on the activated Cu NCs@UiO‐66‐NH_2_ (equilibrium pressure of 6.11 torr).

In order to explain the space‐position‐incurred catalytic activity difference, X‐ray absorption spectroscopy (XAS) studies were carried out to shed light on both the electronic states and local chemical environments of the catalysts. Normalized X‐ray absorption near‐edge structure (XANES) spectra at the Cu K‐edge are presented in Figure 6a. The position of the pre‐edge peaks of both Cu NCs@UiO‐66‐NH_2_ and Cu/UiO‐66‐NH_2_ at 8983 eV clearly suggested that the Cu in the composites was in the metallic Cu^0^ oxidation state, which can be explained due to the existence of ultrasmall Cu nanoparticles. Furthermore, the absence of features after the absorption edge in the XAS spectra of hybrid composites, in contrast to the XANES data of the bulk metal, indicated the absence of fourth or higher‐shell atoms in the nanoclusters, which further demonstrated the presence of the ultrasmall Cu nanoclusters in our composites.^[19]^ A detailed analysis of the derivative XANES spectra (Figure 6b) of the composites indicated that the pre‐edge peak associated with Cu^0^ was slightly shifted to higher energies in both Cu/UiO‐66‐NH_2_ and Cu NCs@UiO‐66‐NH_2_ compared to the references, which suggests that there is a minor contribution of oxidized copper species with an oxidation state of +1 or +2. Consistent with the XANES spectra (Figure 6c), the analysis of extended X‐ray absorption fine structure (EXAFS) demonstrated the presence of a main signal centered at around 2.2 Å for the Cu/UiO‐66NH_2_ composite, corresponding to Cu−Cu distances. Interestingly, this signal was shifted to larger values for Cu NCs@UiO‐66‐NH_2_ (2.4 Å), in agreement with the presence of very small Cu NCs.^[20]^ Moreover, a peak at around 1.5 Å was observed and attributed to the scattering path of Cu−O bond, in agreement with the conclusion from derivative XANES.^[21]^ In conclusion, both the synthesized core–shell composites and the MOF with Cu nanoclusters loaded in the pores have a very similar metal valence, as well as coordination environments. In addition, the XAS results revealed that Cu NCs@MOF‐801and Cu/MOF‐801 presented similar environments (Figures S15–17).

To get a deeper understanding of the high reactivity and selectivity of Cu NCs@UiO‐66‐NH_2_, *in situ* IR spectroscopy was applied in transmission mode on the core–shell composites. NO and CO were used as probe gas molecules to evaluate the different accessible metal sites. As well‐documented in the literature, NO and CO are ideal candidates to selectively detect Cu^2+^, Cu^+^, and Cu^0^ sites, respectively, due to their strong and characteristic interactions with each Cu oxidation state.^[22]^ As shown in Figures S18 and S19, no characteristic NO vibration peak was observed in the range of 1850–1900 cm^−1^ for both composites, which indicated the absence of Cu^2+^ sites.^[22a]^ The stepwise dosing of CO into the cell at room temperature was applied subsequently. As shown in Figure 6d, peaks appeared and increased upon the introduction and dosing of CO in the range between 2109–2140 cm^−1^, which are commonly attributed to the interaction between CO and Cu^I^. The two distinct FT‐IR peaks in the composite could be due to a site heterogeneity; i.e., to slightly different coordination environments around the copper cations. In contrast, the Cu^2+^‐CO complex would provide bands above 2160 cm^−1^ at low temperature,^[23]^ not visible in our spectra. The presence of Cu^0^ carbonyls could also be excluded, since they are supposed to provide bands between 2110 cm^−1^ and 2080 cm^−1^ that were absent from the spectra.^[23]^ These results are consistent with the XANES/EXAFS observations if we assume that the surface Cu sites are oxidized in Cu^I^ upon exposure to traces of oxygen in the solution/air, whilst Cu^0^ is present in the core of the nanoclusters, as is often observed with Cu nanoparticles.^[24]^

The composites with different Cu spatial positions exhibited very similar Cu NCs, with similar size, valence, coordination environment, and quantity. Therefore, it is assumed that their different reactivity is likely due to their different Cu spatial distribution. As shown in Figure S21, the emission caused by pristine UiO‐66‐NH_2_ decreased much more significantly in the case of Cu@UiO‐66‐NH_2_ than for Cu/UiO‐66‐NH_2_. This suggests that in the case of the core–shell composite, the charge transfer is likely to be promoted rather than in the Cu/MOF composite. This is probably due to a more intimate interaction between the Cu nanoparticles and the MOF framework favoring an electron migration, similar to the previous reports for other hybrid composites.^[25]^ Such a synergetic effect is particularly in good line with a few reported similar core–shell composites.[^2^, ^26^]

## Conclusion

In summary, we have developed a new sustainable route to prepare ultrasmall Cu nanoclusters@MOFs (including MOF‐801 and UiO‐66‐NH_2_). The cheap starting materials and facile synthesis approach allowed production of both catalysts at the gram scale for the first time for such MOF‐based core–shell composites. A seed‐mediated growth mechanism was employed, enabling us to acquire insights into the formation mechanism of these core–shell structures. Remarkably, Cu NCs@MOF‐801 exhibited efficient CO_2_ photoreduction rates of 94 μmol h^−1^ g^−1^. Cu NCs@UiO‐66‐NH_2_ showed a 36 % higher catalytic rate than Cu NCs@MOF‐801, and improved selectivity towards formic acid, which highlighted the importance of the polar functional groups on the MOF linker. In addition, the comparison of catalytic efficiencies between Cu NCs on the surface/in the pores of the host MOFs and the core–shell composites revealed a 3 times higher catalytic reactivity for the core–shell architectures. XANES/EXAFS and *in situ* IR spectroscopy measurements were then applied to demonstrate the impact of closer packing of the host/guest in dominating the catalytic reactivity. This new method is not only of interest for synthetic chemists involved in the design of advanced core–shell composites encompassing fragile active compounds, but also of importance for the rational construction of new generations of highly efficient MOF based heterogeneous catalysts.

## Conflict of interest

The authors declare no conflict of interest.

1

## Supporting information

As a service to our authors and readers, this journal provides supporting information supplied by the authors. Such materials are peer reviewed and may be re‐organized for online delivery, but are not copy‐edited or typeset. Technical support issues arising from supporting information (other than missing files) should be addressed to the authors.

Supporting Information

## Data Availability

The data that support the findings of this study are available from the corresponding author upon reasonable request.
