# Supplementary material for: Ultrasmall Copper Nanoclusters in Zirconium Metal‐Organic Frameworks for the Photoreduction of CO2
Source: Angew Chem Int Ed Engl. 2022 Sep 23;61(43):e202211848. doi: 10.1002/anie.202211848 (PMC9826431; doi:10.1002/anie.202211848)
Supplement: Supplementary file 1 — Supporting Information [file ANIE-61-0-s001.pdf]

Angewandte Chemie
Eine Zeitschrift der Gesellschaft Deutscher Chemiker

Supporting Information

Ultrasmall Copper Nanoclusters in Zirconium Metal-Organic Frameworks for the Photoreduction of CO_2

S. Dai, T. Kajiwara, M. Ikeda, I. Romero-Muñiz, G. Patriarche, A. E. Platero-Prats, A. Vimont, M. Daturi, A. Tissot, Q. Xu*, C. Serre**

Materials and methods:

All chemicals were purchased from commercial suppliers and used as received without further purification. Fumaric acid, 98%, Alfa Aesar. 2-Aminoterephthalic acid(BDC-NH_2), 99+%, Acros. 1H-Pyrazole-3,5-dicarboxylic acid(PDA), 97%, FluoroChem. $ZrCl_4$ anhydrous, 98%, Acros. Isopropyl alcohol, 99+%, Sigma. Acetic acid, 99+%, Fisher. Ethanol absolute, >=99%, Acros. L-ascorbic acid, Analytical reagent, Fisher. Copper Nitrate hemipentahydrate, 98%, Acros. Distilled water, Millipore system.

Powder X-ray Diffraction (PXRD) data were recorded on a high-throughput Bruker D8 Advance diffractometer working on transmission mode and equipped with a focusing Göbel mirror producing Cu Kα radiation (λ = 1.5418 Å) and a LynxEye detector. Nitrogen porosimetry data were collected on a Micromeritics Tristar/ Triflex instrument at 77 K (pre-activating samples at 100 °C under vacuum, 12 hours). Scanning Electron Microscopy coupled with energy-dispersive X-ray spectroscopy (SEM-EDX) results were recorded with FEI Magellan 400 scanning electron microscope. Thermogravimetric analysis (TGA) data were collected on Mettler Toledo TGA/DSC 2, STAR System apparatus with a heating rate of 5 °C/min under the oxygen flow. Infrared spectra were measured with a Nicolet iS5 FTIR ThermoFisher spectrometer. Zeta potential and DLS size measurements of hydrodynamic radii were made on a Malvern Zetasizer Nano-ZS (Malvern Instruments). ICP-MS was performed with an Agilent 7850 elemental analyzer. High resolution TEM images (HRTEM) were acquired on a Titan Themis 200 microscope operating at 200 kV. This microscope is equipped with a Ceta 16M hybrid camera from ThermoFischer Scientific capable of working under low electron irradiation conditions. The HRTEM images were obtained in low dose condition with an irradiation current between 100 and 250 electrons per square angstroms. *In situ* infrared spectroscopy measurements were conducted in a homebuilt quartz cell equipped with KBr windows. A movable quartz sample holder permits the adjustment of the pellet in the infrared beam for spectra acquisition and to displace it into a furnace at the top of the cell for thermal treatments. The samples were pressed (100 bar) into a self-supported discs (2 cm^2 area, 7-10 mg cm^{-2}). IR spectra were recorded in transmission mode at 4 cm^{-1} resolution on a Nicolet Nexus spectrometer equipped with an extended KBr beam splitting device and a mercury cadmium telluride (MCT) cryo-detector, and each spectrum presented was an average of 256 scans. Synchrotron XANES and EXAFS measurements were investigated in ALBA, Barcelona, Spain. ^{13}C NMR (Water-d_2, 2048 merged scans) was performed using 600 MHz NMR spectrometer Bruker.

X-ray absorption spectroscopy: Transmission and fluorescence geometry XAS measurements were performed at CLAESS (BL22) at the ALBA synchrotron. Cu K-edge XAS spectra were acquired from 8868 to 9500 eV at 20 K, resulting in a *k*-range up to 12 $Å^{-1}$. Data analysis and background removal were performed using ATHENA program from the DEMETER software package.[1] The radial values are without phase correction. Cu foil, CuO, CuCl and Cu foil were employed as references.

CO_2 reduction: To a glass test tube was added a catalyst (10 mg), triethanolamine (TEOA, 0.6 mL), and *N,N*-dimethylacetamide (DMA, 9.4 mL). The volumes of gas and liquid phases were 53.5 and 10.0 mL, respectively. The tube was capped with a silicone septum and degassed with CO_2 by bubbling through the reaction mixture (150 mL/min, 15 min). The CO_2-saturated suspension was stirred and irradiated using a 300 W Xe lamp (MAX-303 equipped with UV (250–385 nm) mirror module, Asahi Spectra) at room temperature for 18 h. The amounts of CO, CH_4, and H_2 in the gas phase were determined by a gas

chromatograph equipped with a thermal conductivity detector (GC-2014, SHIMADZU). The amount of CH_3OH in the liquid phase was determined by a gas chromatograph mass spectrometer (GCMS-QP2010 Ultra, SHIMADZU). The amount of HCOOH in the liquid phase was determined by a liquid chromatograph equipped with a conductivity detector (Prominence Organic Acid Analysis System, SHIMADZU). The amount of HCHO in the liquid phase was determined by a liquid chromatograph mass spectrometer equipped with a diode array detector (LCMS-2020, SHIMADZU) after the treatment with 2,4-dinitrophenylhydrazine (DNPH).

Synthesis of Cu NCs: L-ascorbic acid (880 mg, 5 mmol) was added to $Cu(NO_3)_2$*$2.5H_2O$ (116 mg, 0.5 mmol) in 55 mL aqueous solution with strong stirring. The solution was allowed to stir at room temperature for 1.5 h. The resulting light-yellow solution was used throughout the following experiments.

Synthesis of Zr_6 oxoclusters: The synthesis of Zr_6 oxolcusters followed the step similar to the reported article.[2] 10g $ZrCl_4$ was added into a mixture of 15 mL of glacial acetic acid and 25 mL of isopropanol under stirring at 600 rpm and heated at 120 °C for 60 min. The product was collected either through suction filtration or centrifugation at 10,000 rpm. The collected white solid was subsequently washed with acetone twice and dried under vacuum at room temperature.

Synthesis of Cu NCs@MOF-801 (with 6.4 mmol Cu NCs): Zirconium oxoclusters ($Zr_6O_4(OH)_4(C_2H_3O_2)_8Cl_4$, 1 g) were stirred in acetic acid (20 mL, 5 mol/L) until well dispersed. Then, an aqueous solution of Cu NCs (50 mL) was added. The mixture was kept stirring for a few minutes until all Zr_6 oxoclusters were dissolved. Then, Fumaric acid (532 mg, 4.6 mmol) was added and the resulted mixture was kept stirring for 3 days until observation of cloudy yellowish solution. The solid was collected and washed 5 times with only H_2O. Final solids were then dried under vacuum.

Synthesis of Cu NCs@UiO-66-NH_2: Zirconium oxoclusters ($Zr_6O_4(OH)_4(C_2H_3O_2)_8Cl_4$, 1.2 g) were stirred in acetic acid (8 mL, 3.5 mol/L) until well dispersed. Then, an aqueous solution of Cu NCs (20 mL) was added. The mixture was kept stirring for a few minutes until all Zr_6 oxoclusters were dissolved. Then, 2-aminoterephthalic acid (400 mg, 2.2 mmol) and 20 mL of ethanol were added. The resulted mixture was kept stirring for 3 days until observation of cloudy yellowish solution. The solid was collected and washed 5 times with only H_2O. Final solids were then dried under vacuum.

Synthesis of Cu/MOF-801: 76 mg of well-washed and dry MOF-801 powder was immersed in 4 mL H_2O. 0.2 mL 0.1M $Cu(NO_3)_2$ was introduced to the solution with sonication to make it homogeneous. Subsequently, 35 mg L-ascorbic acid was added into solution with strong stirring for 12h. The collected composites were washed simply with water for 2 times and dried under vacuum at room temperature.

Synthesis of Cu/UiO-66-NH_2: 76 mg of well-washed and dry UiO-66-NH_2 powder was immersed in 4 mL H_2O. 0.2 mL 0.1M $Cu(NO_3)_2$ was introduced to the solution with sonication to make it homogeneous. Subsequently, 35 mg L-ascorbic acid was added into solution with strong stirring for 12h. The collected composites were washed simply with water for 2 times and dried under vacuum at room temperature.

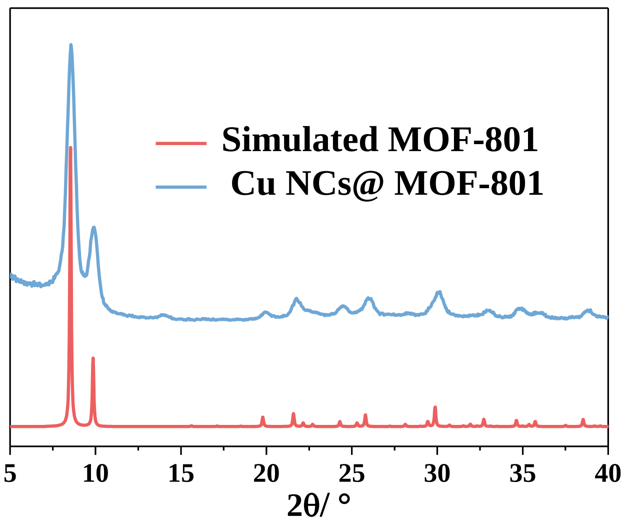

Figure S1. PXRD patterns of Cu NCs@MOF-801 and the simulated one.

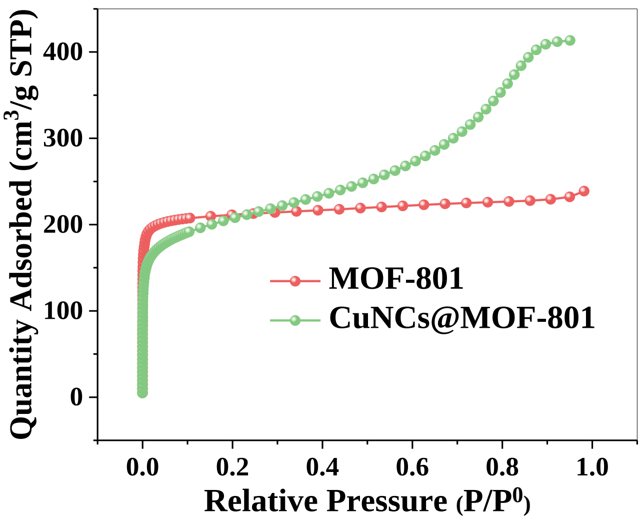

Figure S2. 77 K N_2 isotherms of pristine MOF-801 and Cu NCs@MOF-801.

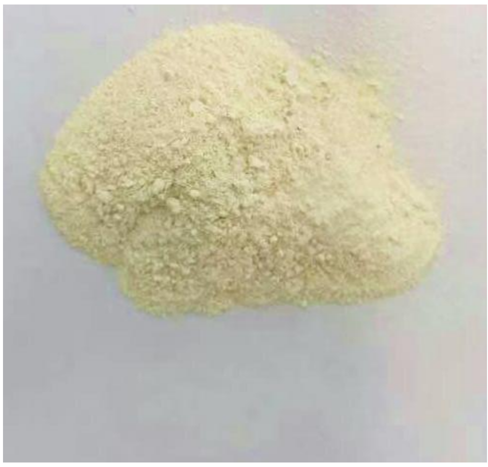

Figure S3. 1 g of Cu NCs@MOF-801.

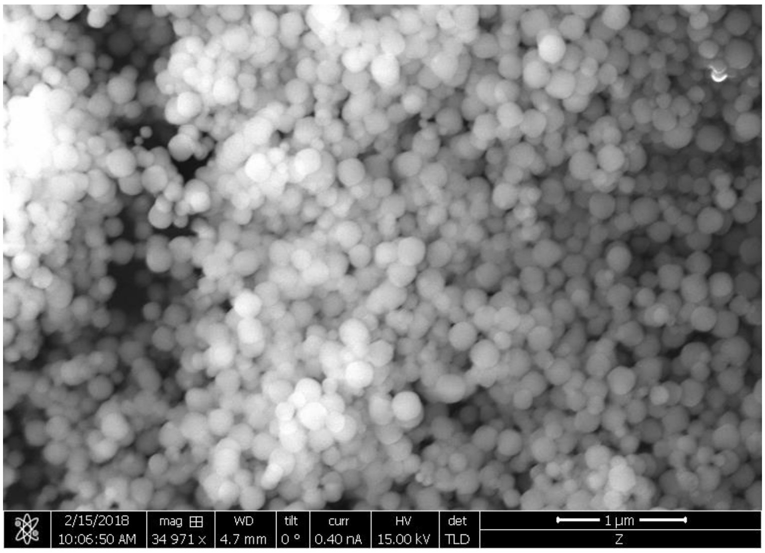

Figure S4. SEM image of MOF-801 prepared without Cu NCs, which corresponds to the PXRD pattern in Figure 2d(1).

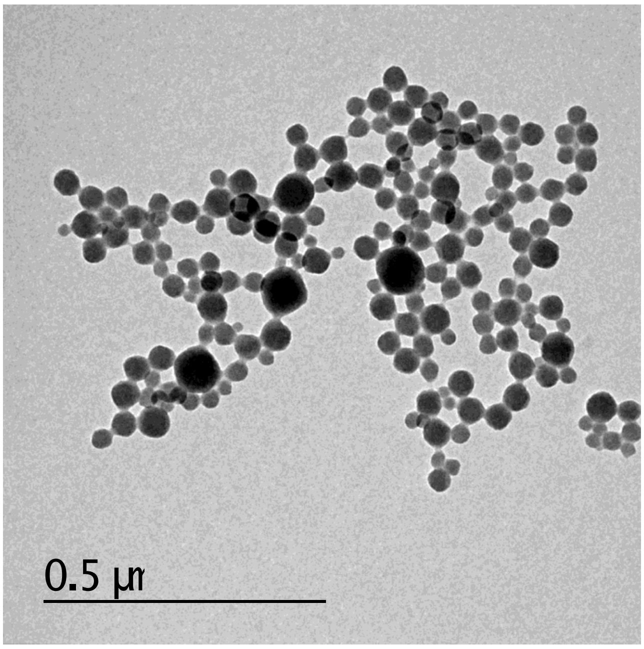

Figure S5. TEM image of Cu NCs@MOF-801 prepared with 64 μmol Cu NCs, which corresponds to the PXRD pattern in Figure 2d(3).

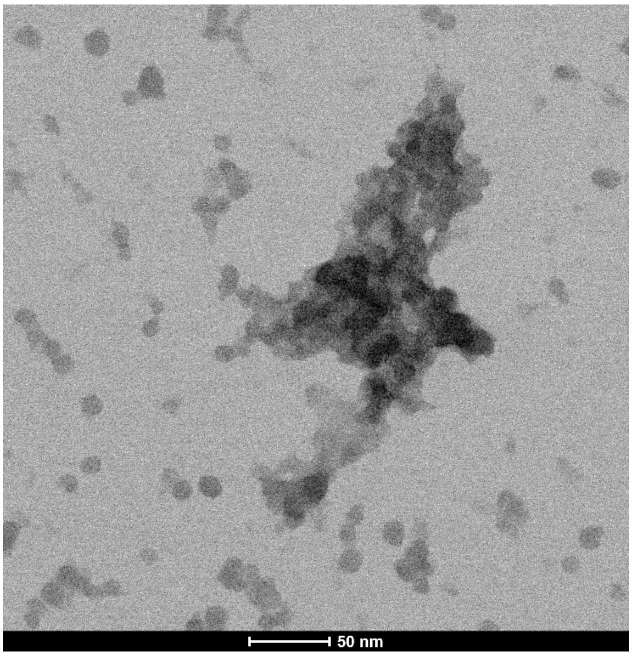

Figure S6. TEM image of Cu NCs@MOF-801 prepared with 6.4 mmol Cu NCs, which corresponds to the PXRD pattern in Figure 2d(9).

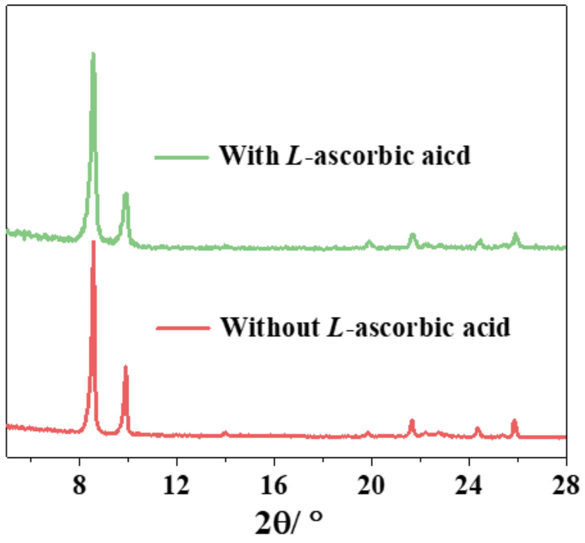

Figure S7. PXRD patterns of MOF-801 synthesized with and without L-Ascorbic acid.

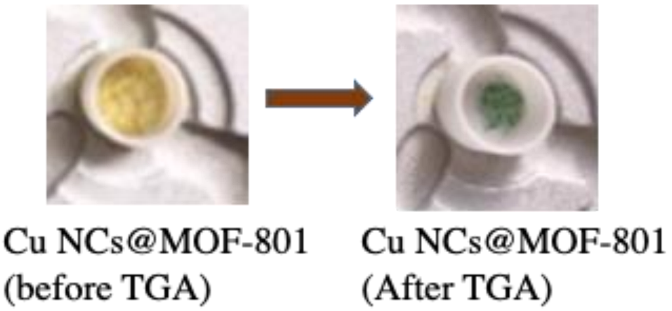

Figure S8. Photograph of Cu NCs@MOF-801 before and after TGA measurements under O_2.

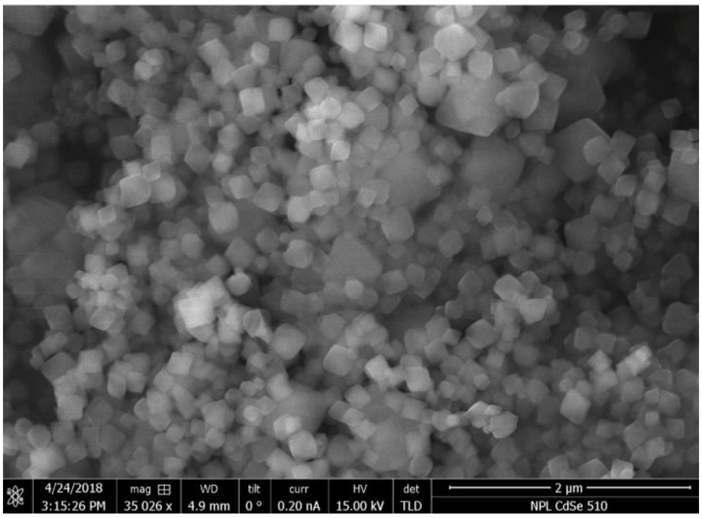

Figure S9. SEM image of UiO-66-NH_2 nanocrystals synthesized without the addition of Cu NCs.

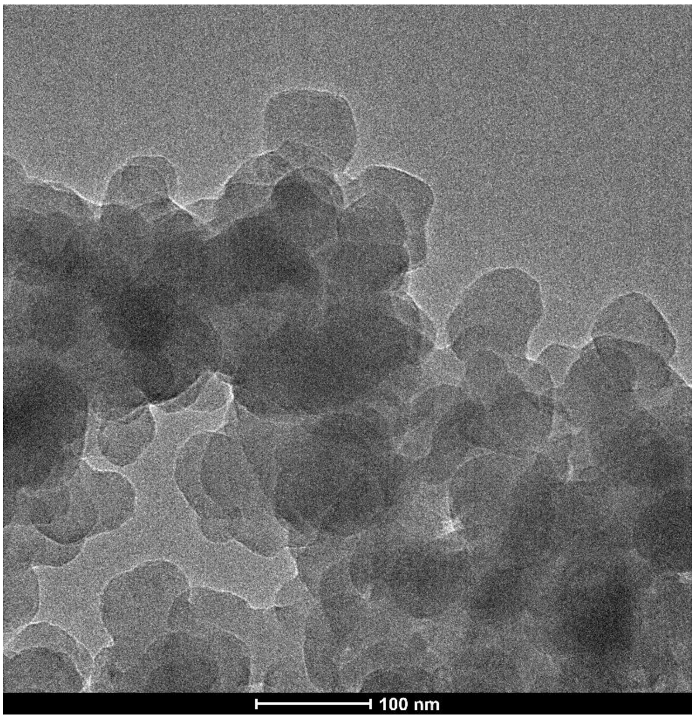

Figure S10. TEM image of UiO-66-NH_2 synthesized with 20 mL, 6.4 mmol Cu NCs.

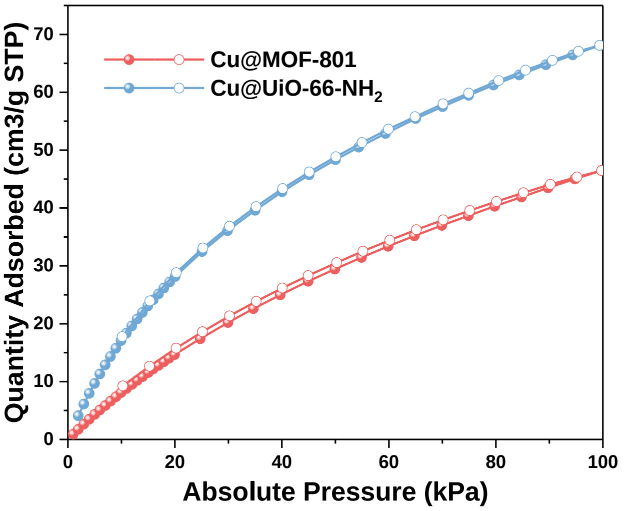

Figure S11: 298K CO_2 adsorption/desorption of Cu NCs@MOF-801, and Cu NCs@UiO-66-NH_2.

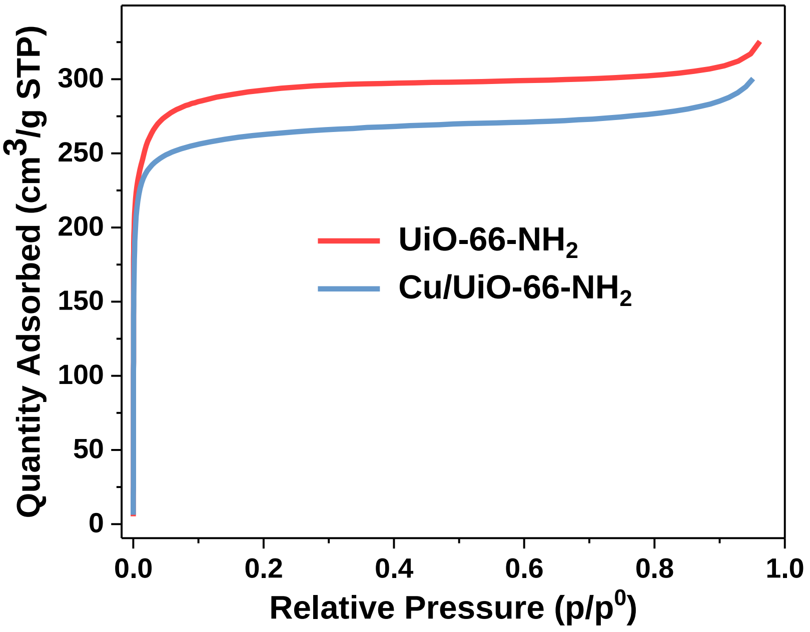

Figure S12. 77K N_2 adsorption of Cu/UiO-66-NH_2 in comparison to the pristine UiO-66-NH_2.

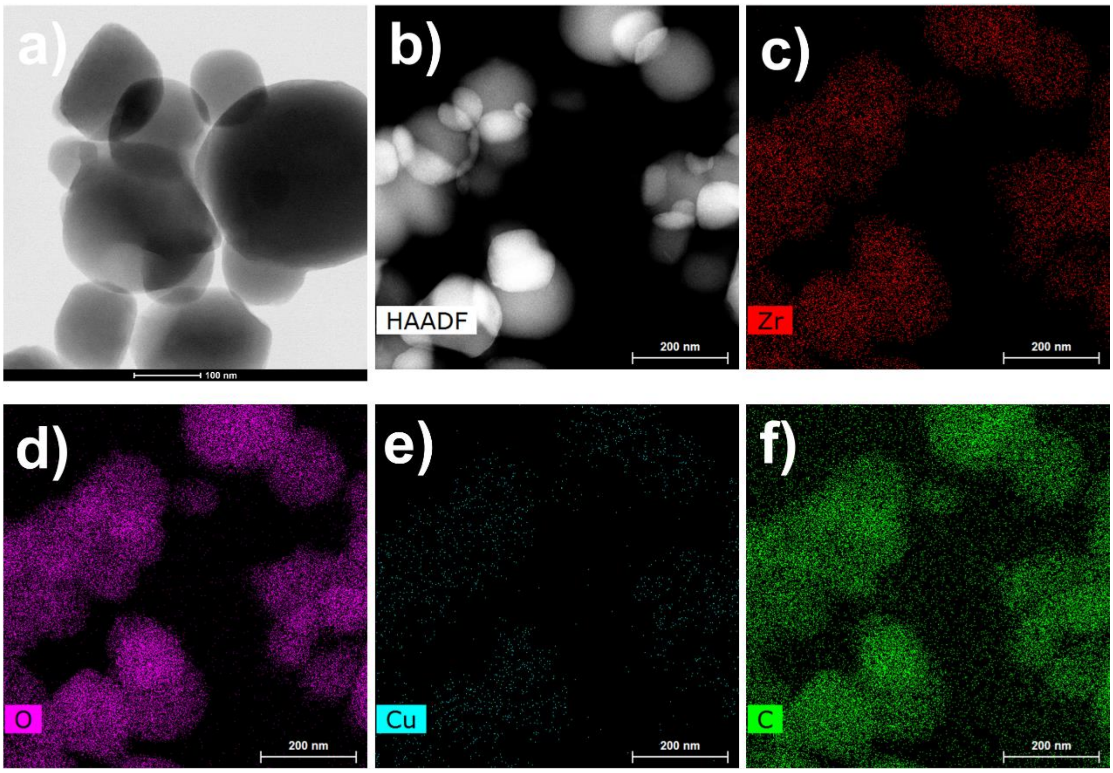

Figure S13. Electron microscope characterizations of Cu/MOF-801, a) high-resolution TEM image, b) HAADF-STEM image, c-f) EDS mapping of Zr, O, Cu, and C.

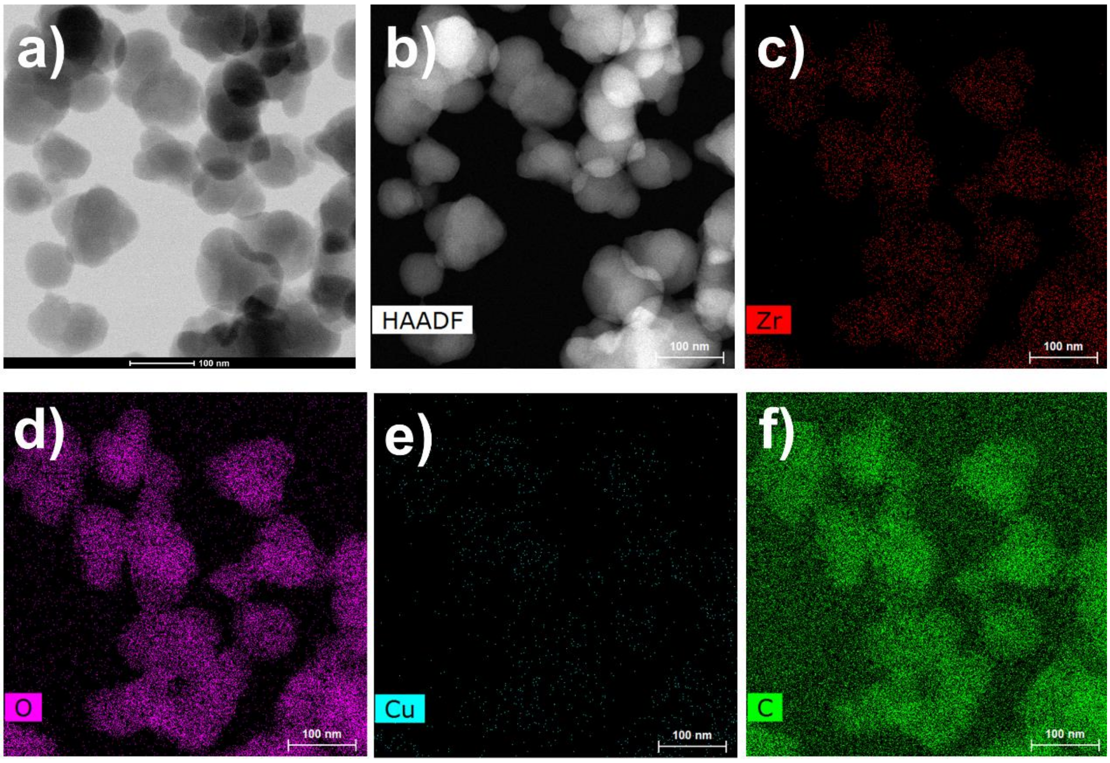

Figure S14. Electron microscope characterizations of Cu/UiO-66-NH_2, a) high-resolution TEM image, b) HAADF-STEM image, c-f) EDS mapping of Zr, O, Cu, and C.

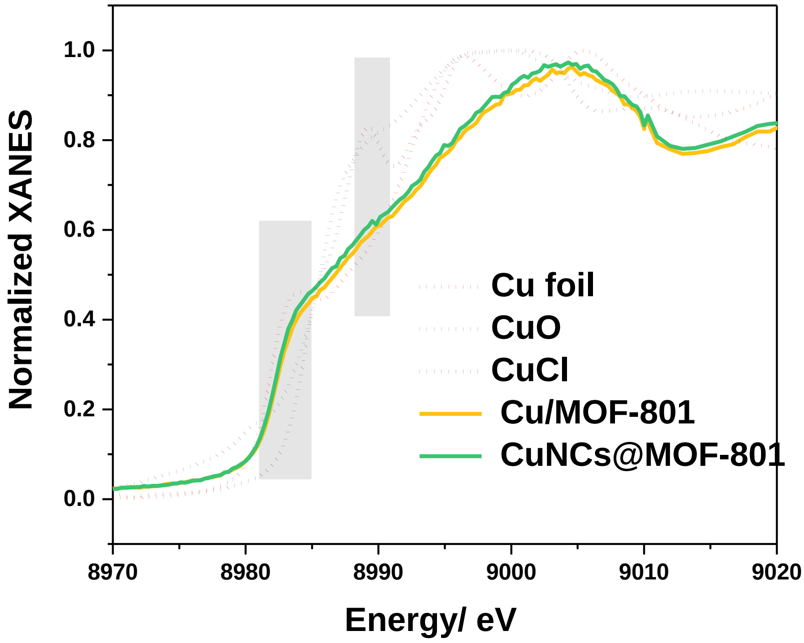

Figure S15. Cu K-edge XANES of Cu NCs@MOF-801, Cu/MOF-801 and the references (Cu foil, CuO and CuCl).

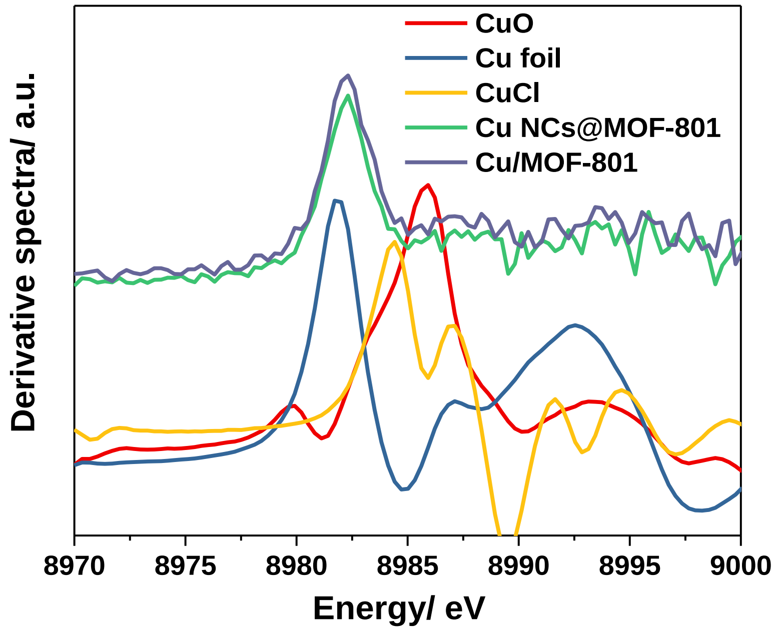

Figure S16. Cu K-edge derivative XANES of Cu NCs@MOF-801, Cu/MOF-801 and references (Cu foil, CuO and CuCl).

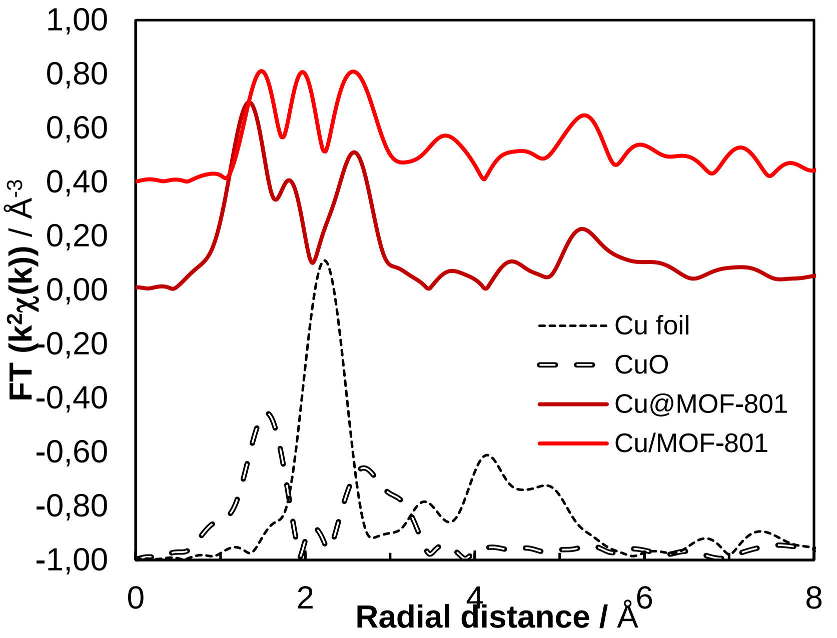

Figure S17. Fourier-transformed EXAFS spectra of Cu NCs@MOF-801, Cu/MOF-801 and references (Cu foil, CuO and CuCl).

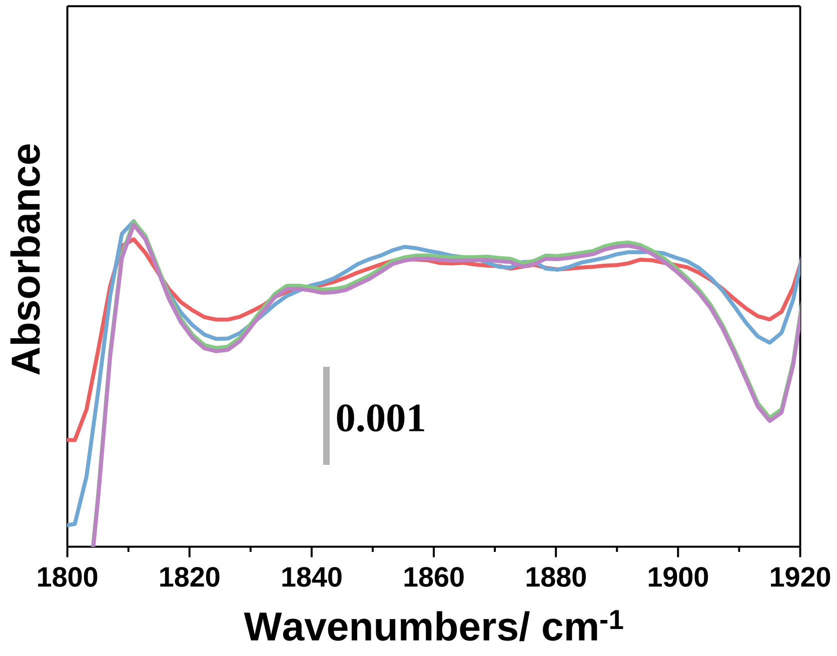

Figure S18. FTIR spectra of NO adsorption of Cu NCs@UiO-66-NH_2 at room temperature, small doses up to 5.3 torr.

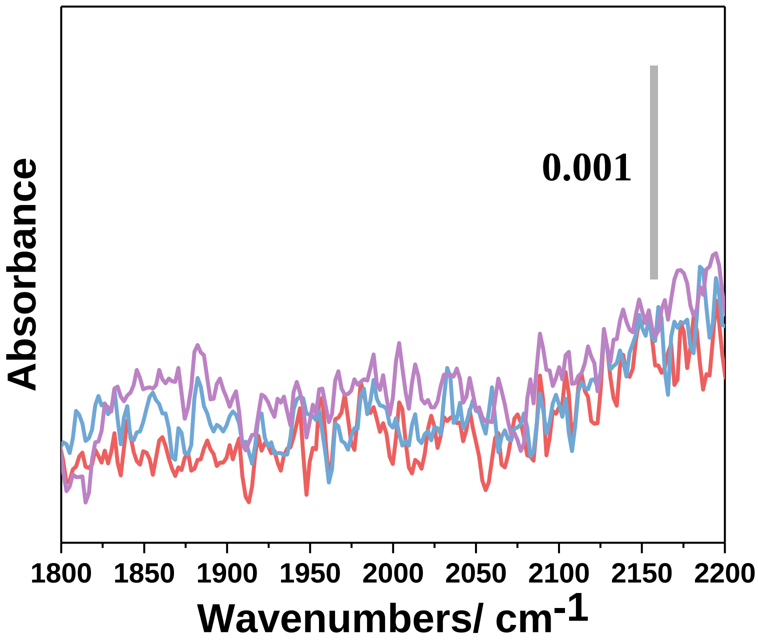

Figure S19. FTIR spectra of NO adsorption at room temperature of Cu NCs@MOF-801, small doses up to 4.2 torr.

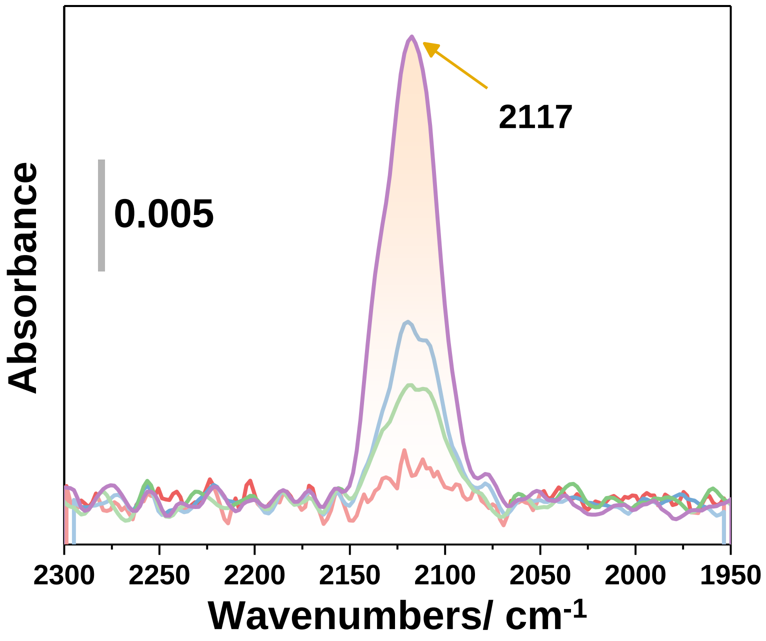

Figure S20. FTIR spectra of CO adsorption at room temperature of Cu NCs@MOF-801, small doses up to equilibrium pressure of 5.69 torr.

Table 1. Summarized the formate production of some MOFs or MOFs-based composites.

Materials	Catalytic medium and electrolyte	Light source	Formic acid Production/ selectivity ($\mu mol \cdot h^{-1} \cdot g^{-1}$)	Ref.
MIL-125(Ti)-NH_2	Aqueous suspension in MeCN and TEOA	500W Xe lamp	16.2	[3]
MIL-125(Ti)			4.28	[3]
UiO-66-NH_2	Aqueous suspension in MeCN and TEOA	500W Xe lamp	26.4	[4]
MIL-88(Fe)	MeCN/TEOA(5:1)	300W Xe lamp	22.5	[5]
MIL-88-NH_2(Fe)			75	[5]
MIL-125-NHMe	TEOA (0.3M) mesitylene (0.0015M) in MeCN-d_3	Blue light λ=466 nm (LED light source)	0.284	[6]
PCN-222	MeCN/TEOA(10:1)	300W high pressure Hg lamp	60	[7]
NNU-28	MeCN/TEOA(30:1)	300W Xe lamp	52.8	[8]
UiO-66-NH_2 (Zr/Ti)	MeCN/TEOA (4:1) BNAH (0.1M)	300W Xe lamp	4.66±0.17	[9]
UiO-66-$(NH_2)_2$ (Zr/Ti)			6.27±0.23	[9]
$\{Cd_2[Ru(4,4'\text{-dcbpy})_3]\cdot 12H_2O\}n$ nanoflower	MeCN/TEOA(20:1)	500W Xe lamp	77.2	[10]
$\{Cd_2[Ru(4,4'\text{-dcbpy})_3]\cdot 12H_2O\}n$ Nano-flakes			52.7	[10]
$\{Cd_2[Ru(4,4'\text{-dcbpy})_3]\cdot 12H_2O\}n$ Bulk crystals			30.6	[10]
Pt/MIL-125(Ti)-NH_2	MeCN/TEOA (5:1)	300W Xe lamp	32.4	[11]
Au/ MIL-125(Ti)-NH_2			16.3	[11]
**Cu NCs@MOF-801**	**TEOA/DMA**	**300W Xe lamp**	**94**	**This work**
**Cu NCs@UiO-66-NH_2**			**128**	**This work**

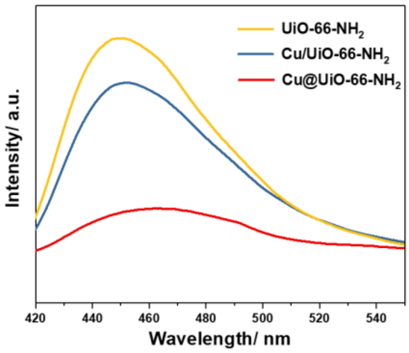

Figure S21. Photoluminescence spectrum of UiO-66-NH_2, Cu/UiO-66-NH_2, and Cu NCs@UiO-66-NH_2, E_λ=395 nm.

1. Ravel, B.; Newville, M., ATHENA, ARTEMIS, HEPHAESTUS: data analysis for X-ray absorption spectroscopy using IFEFFIT. *J. Synchrotron Radiat.* **2005,** *12* (4), 537-541.

2. Dai, S.; Simms, C.; Dovgaliuk, I.; Patriarche, G.; Tissot, A.; Parac-Vogt, T. N.; Serre, C., Monodispersed MOF-808 Nanocrystals Synthesized via a Scalable Room-Temperature Approach for Efficient Heterogeneous Peptide Bond Hydrolysis. *Chem. Mater.* **2021,** *33* (17), 7057-7066.

3. Fu, Y.; Sun, D.; Chen, Y.; Huang, R.; Ding, Z.; Fu, X.; Li, Z., An amine-functionalized titanium metal–organic framework photocatalyst with visible-light-induced activity for CO2 reduction. *Angew. Chem., Int. Ed.* **2012,** *51* (14), 3364-3367.

4. Sun, D.; Fu, Y.; Liu, W.; Ye, L.; Wang, D.; Yang, L.; Fu, X.; Li, Z., Studies on Photocatalytic CO2 Reduction over NH2-Uio-66 (Zr) and Its Derivatives: Towards a Better Understanding of Photocatalysis on Metal–Organic Frameworks. *Chem. Eur. J.* **2013,** *19* (42), 14279-14285.

5. Wang, D.; Huang, R.; Liu, W.; Sun, D.; Li, Z., Fe-based MOFs for photocatalytic CO2 reduction: role of coordination unsaturated sites and dual excitation pathways. *Acs Catal.* **2014,** *4* (12), 4254-4260.

6. Logan, M. W.; Ayad, S.; Adamson, J. D.; Dilbeck, T.; Hanson, K.; Uribe-Romo, F. J., Systematic variation of the optical bandgap in titanium based isoreticular metal–organic frameworks for photocatalytic reduction of CO 2 under blue light. *J. Mat. Chem. A* **2017,** *5* (23), 11854-11863.

7. Xu, H.-Q.; Hu, J.; Wang, D.; Li, Z.; Zhang, Q.; Luo, Y.; Yu, S.-H.; Jiang, H.-L., Visible-light photoreduction of CO2 in a metal–organic framework: boosting electron–hole separation via electron trap states. *J. Am. Chem. Soc.* **2015,** *137* (42), 13440-13443.

8. Chen, D.; Xing, H.; Wang, C.; Su, Z., Highly efficient visible-light-driven CO 2 reduction to formate by a new anthracene-based zirconium MOF via dual catalytic routes. *J. Mat. Chem. A* **2016,** *4* (7), 2657-2662.

9. Lee, Y.; Kim, S.; Kang, J. K.; Cohen, S. M., Photocatalytic CO 2 reduction by a mixed metal (Zr/Ti), mixed ligand metal–organic framework under visible light irradiation. *Chem. Commun.* **2015,** *51* (26), 5735-5738.

10. Zhang, S.; Li, L.; Zhao, S.; Sun, Z.; Hong, M.; Luo, J., Hierarchical metal–organic framework nanoflowers for effective CO2 transformation driven by visible light. *J. Mat. Chem. A* **2015,** *3* (30), 15764-15768.

11. Sun, D.; Liu, W.; Fu, Y.; Fang, Z.; Sun, F.; Fu, X.; Zhang, Y.; Li, Z., Noble metals can have different effects on photocatalysis over metal–organic frameworks (MOFs): a case study on M/NH2-MIL-125 (Ti)(M= Pt and Au). *Chem. Eur. J.* **2014,** *20* (16), 4780-4788.
